# How can a high-quality genome assembly help plant breeders?

**DOI:** 10.1093/gigascience/giz068

**Published:** 2019-06-10

**Authors:** Juliana Benevenuto, Luís Felipe V Ferrão, Rodrigo R Amadeu, Patricio Munoz

**Affiliations:** Blueberry Breeding and Genomics Laboratory, Horticultural Sciences Department, University of Florida, Gainesville, 2550 Hull Road, FL, USA

**Keywords:** Vaccinium, genome assembly, gene, GWAS, genomic prediction

## Abstract

The decreasing costs of next-generation sequencing and the improvements in *de novo* sequence assemblers have made it possible to obtain reference genomes for most eukaryotes, including minor crops such as the blueberry (*Vaccinium corymbosum*). Nevertheless, these genomes are at various levels of completeness and few have been anchored to chromosome scale and/or are haplotype-phased. We highlight the impact of a high-quality genome assembly for plant breeding and genetic research by showing how it affects our understanding of the genetic architecture of important traits and aids marker selection and candidate gene detection. We compared the results of genome-wide association studies and genomic selection that were already published using a blueberry draft genome as reference with the results using the recent released chromosome-scale and haplotype-phased blueberry genome. We believe that the benefits shown herein reinforce the importance of genome assembly projects for other non-model species.

## Background

Assembling plant genomes using short-read–based sequencing is a challenging task, especially because most plant genomes are large, highly repetitive, and have undergone ancient and recent rounds of polyploidization. Thanks to the new sequencing methods, researchers have been able to achieve chromosome-scale haplotype-phased genome assemblies more inexpensively and quickly than in previous decades. The cultivated blueberry (*Vaccinium corymbosum*) is an outcrossing tetraploid species (2n = 4X = 48), and 48 pseudomolecules from the northern highbush cultivar 'Draper' were recently assembled and phased [1]. To accomplish this, the authors used a combination of Illumina paired-end and mate-pair libraries, 10X Genomics Chromium, and Hi-C scaffolding strategies.

**Figure 1:**
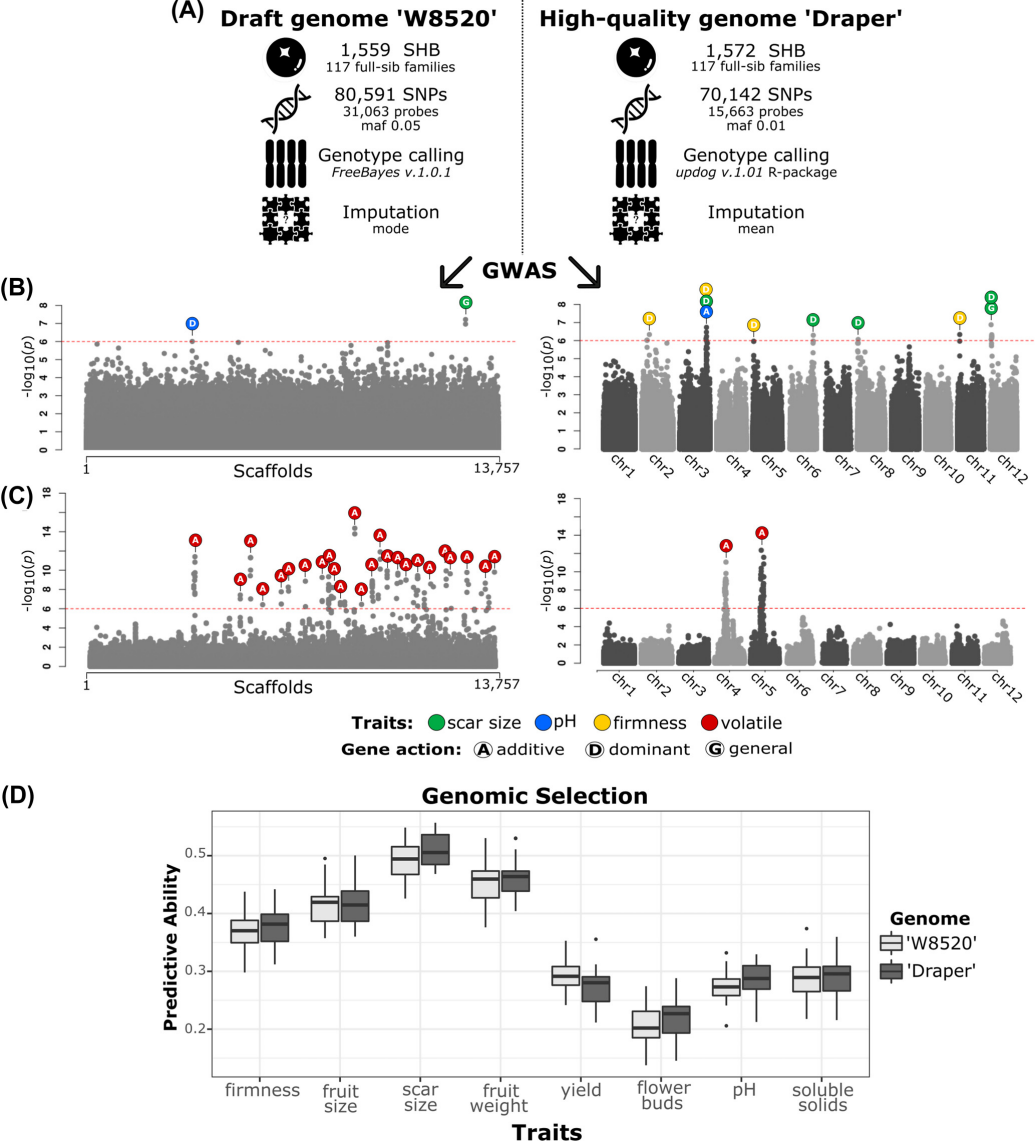
**(A)** Differences in plant material and analytical pipeline using the draft genome 'W8520' and the chromosome-scale haplotype-phased genome 'Draper.' Additional steps not mentioned in this figure were performed according to Ferrão et al. [2]. **(B)** GWAS analyses performed for fruit-related traits (scar size, pH, and firmness) using both genome assemblies and considering Bonferroni threshold of 0.05. **(C)** GWAS analyses performed for the volatile geranyl-acetone (CAS 3796–70-1) for individuals from the same SHB population (unpublished data by Patricio Munoz), using both genome assemblies and considering Bonferroni threshold of 0.05. **(D)** Predictive abilities for 8 blueberry fruit-related traits using 31,000 probes in the 'W8520' draft genome and using 15,000 selected probes using the 'Draper' genome as reference. For genomic prediction, we used Genomic Best Linear Unbiased Prediction (GBLUP) implemented in the sommer R package, considering tetraploid inheritance in the AGH-matrix R package, and 30-fold cross-validation by splitting the population into 70% training and 30% testing. chr: chromosome; maf: minor allele frequency.

One year ago, our group at University of Florida performed genome-wide association study (GWAS) analyses in a southern highbush blueberry (SHB) breeding population in order to detect single-nucleotide polymorphisms (SNPs) associated with fruit-related traits [2]. At that time, we used the available draft genome as a reference for SNP calling and gene mining of significant associations. The draft genome assembly was performed for a diploid northern highbush ('W8520') using short-reads from 454 pyrosequencing and Illumina platforms [3, 4]. This draft assembly is highly fragmented, with 13,757 scaffolds (N50 of 145 kb) and incomplete gene predictions. With the recent release of a new genome assembly by Colle et al. at Michigan State University [1], we raised the question: how would a high-quality reference genome affect our previous results and future research? To this end, we re-analyzed our data using nearly the same SHB breeding population, but accommodating changes in probe selection and tetraploid genotype calling that, currently, we believe to be more appropriate (see Fig. 1A). The impact of a chromosome-scale and haplotype-phased genome was compared in terms of unique probe alignment, genetic architecture of the traits, candidate gene mining, and genomic prediction.

## Selection of Probes for Targeted SNP Calling

A total of 31,063 probes of 120-mer were originally designed based on the 'W8520' genome for targeted Capture-Seq genotyping by RAPiD Genomics (Gainesville, FL, USA). Probes were designed for enrichment of genic and single mapped genomic regions. The probe sequences were then aligned against the high-quality 'Draper' genome using blastn with e-value threshold of 10^−10^ and identity of 80% [5]. Because the genome assembly is haplotype-phased, we were able to distinguish probes that aligned only within homeologous groups but not among them. Therefore, the new genome allowed us to better filter uniquely mapped probes, and only half of the original probes (15,663) were further used for targeted SNP calling of the SHB population (Fig. 1A). The largest chromosome of each homeologous set (12 total) from the 'Draper' genome was used as reference for SNP calling, as described by Ferrão et al. [2]. Likewise, a high-quality reference genome also refines the proportion of uniquely mapped reads during the alignment of resequencing data as shown in *Brassica* species [6].

## Genetic Architecture of the Traits

GWAS can provide the first insights into the genetic architecture of a trait by identifying the number of significant loci, genomic position, mode of gene action, and effect on the phenotypic variation. In this step, the high-resolution positioning of SNPs in the chromosome-scale assembly played an important role in unraveling the genetic architecture of the traits. Using this new pipeline, which also included more accurate genotype calling using the updog R package [7], we were able to find significant SNPs with additive gene action mode (e.g., fruit pH), and novel associations (e.g., fruit firmness and scar size) that were not detected in our previous publication [2] (Fig. 1B). Moreover, we performed GWAS for a new trait, the volatile geranyl-acetone, extracted and quantified using gas chromatography−mass spectrometry, for individuals in the same SHB population. Using the 'W8520' genome as reference, the significant SNPs for this volatile were scattered throughout the unplaced scaffolds, leading to a mistaken interpretation that many loci are involved in the trait variation (i.e., polygenic). When the high-quality 'Draper' genome was used, the significant SNPs converged to a tower-like structure in the Manhattan plot (Fig. 1C), indicating that, instead of polygenic, there are most likely 2 genomic regions contributing for the trait variation (i.e., oligogenic).

## Candidate Gene Mining

GWAS also provides candidate genes for subsequent validation. A high-quality genome assembly results in a more complete and accurate prediction of the gene repertoire for candidate gene mining. To exemplify, we looked at the nearest gene of the associated SNPs mentioned in Fig. 2A for scar size and pH traits in the 'W8520' genome. Both predicted sequences (*CUFF.54762.1* and *CUFF.14779.1*) were incomplete, and no significant similarity was found in the non-redundant blast protein database. However, for all the nearest genes predicted in the 'Draper' genome, we could find orthologs and/or functional annotation (Supplementary Table). Similarly, an improved assembly version of the wheat genome (*Triticum aestivum* L.) allowed the full resolution of a quantitative trait locus (QTL) in a region that was disrupted in the previous wheat assemblies due to lack of ordering and incomplete gene predictions [8]. In addition, a more complete annotation of genome features provided a framework for selecting targets and design guide RNAs for editing genes underlying traits relevant for breeding [8].

## Genomic Selection

Genomic selection (GS) has become a new tool in breeding programs, assisting the selection of promising materials and maximizing the genetic gains. For its implementation, a high marker density is required in order to capture most of the linkage information between QTLs and SNPs. However, many studies have been showing that improvements in prediction accuracies reach a plateau afterwards despite the increased marker density [9]. Moreover, targeted genotyping costs are driven by the number of probes and the number of flow-cell lanes to sequence the entire assay. Therefore, finding an optimal balance between the number of probes/markers and predictive ability is important for a cost-effective GS implementation. By using the 'Draper' genome, we were able to halve the number of probes and still achieve similar predictive abilities for most traits compared with the original number in the 'W8520' genome (Fig. 1D). Similar predictive abilities in the same SHB population were also reported by de Bem Oliveira et al. [10] using the 'W8520' genome.

## Conclusions

Altogether, we can conclude that investing time and resources to obtain a high-quality reference genome is worthwhile given the benefits it confers to downstream genetic analyses and in the decision-making process for breeding programs. In the case of the blueberry, the benefits were as follows: (i) selection of a superior set of uniquely mapped probes for GWAS and GS, which will help reduce future targeted genotyping costs because fewer probes are needed; (ii) a higher precision about the location, number, and gene action of QTLs, thereby improving our understanding of the genetic architecture of the traits through GWAS analyses; (iii) higher chances to find the molecular mechanisms underpinning the trait variation in future studies by being able to explore a more complete gene repertoire; and (iv) achievement of similar genomic predictive ability with fewer genotyping probes. All this will translate into less time and funds needed to implement marker-assisted and genomic selection in the breeding program, and hopefully the achievement of higher genetic gains in shorter breeding cycles.

## Availability of supporting data and materials

The data used herein were mostly retrieved from published articles. Specifically, the phenotypic and genotypic data from the SHB population can be obtained from Ferrão et al. [2] at Dryad Digital Repository (doi: 10.5061/dryad.kd4jq6h). The 'Draper' genome from Colle et al. [1] can be downloaded from the CoGe platform (https://genomevolution.org/coge/GenomeInfo.pl?gid=36464) and *GigaScience* database (doi: 10.5524/100537) . The 'W8520' genome from Bian et al. [3] and Gupta et al. [4] can be downloaded from the QuickLoad site (http://www.igbquickload.org/blueberry/V_corymbosum_scaffold_May_2013/).

## Additional files

SupTable_gene_mining_old_newgenome.xlsx.

## Abbreviations

GBLUP: Genomic Best Linear Unbiased Prediction; GS: genomic selection; GWAS: genome-wide association study; kb: kilobase pairs; maf: minor allele frequency; QTL: quantitative trait locus; SNP: single-nucleotide polymorphism; SHB: southern highbush blueberry.

## Competing interests

The authors declare that they have no competing interests.

## Funding

This work was funded by the University of Florida royalty fund generated by the licensing of blueberry cultivars.

## Authors’ contributions

J.B. performed the probe selection, SNP calling, and annotation of the genes. L.F.V.F. performed the GWAS analyses. R.R.A. performed the GS analyses. P.M. supervised and provided overall guidance. J.B. wrote the manuscript with revision from all authors. All authors read and approved the final manuscript.

## Supplementary Material

giz068_GIGA-D-19-00128_Original_Submission

giz068_GIGA-D-19-00128_Revision_1

giz068_Response_to_Reviewer_Comments_Original_Submission

giz068_Reviewer_1_Report_Original_Submission Robert Henry -- 4/23/2019 Reviewed

giz068_Supplemental_File
