# Supplementary material for: How can a high-quality genome assembly help plant breeders?
Source: Gigascience. 2019 Jun 10;8(6):giz068. doi: 10.1093/gigascience/giz068 (PMC6558523; doi:10.1093/gigascience/giz068)
Supplement: giz068_GIGA-D-19-00128_Revision_1 [file giz068_giga-d-19-00128_revision_1.pdf]

GigaScience

How can a high-quality genome assembly help plant breeders?

--Manuscript Draft--

**Manuscript Number:**	GIGA-D-19-00128R1
**Full Title:**	How can a high-quality genome assembly help plant breeders?
**Article Type:**	Commentary
**Funding Information:**	
**Abstract:**	The ever-decreasing costs to generate next-generation sequencing data and the significant improvements in de novo sequence assemblers have made it possible to obtain reference genomes for most eukaryotes, including relatively minor crops such as blueberry (Vaccinium corymbosum). Nevertheless, these genomes are at various levels of completeness and only few have been anchored to chromosome-scale and/or are haplotype-phased. This commentary highlights the impact of a high-quality genome assembly for plant breeding and genetic research by showing how it affects our understanding of the genetic architecture of important traits, aids the marker selection, and the detection of candidate genes. To this end, we compared the results of genome-wide association studies (GWAS) and genomic selection (GS) that were already published using a blueberry draft genome as reference with the results using the recent released chromosome-scale and haplotype-phased blueberry genome. Although this comparison had been motivated by its potential application in blueberry, we believe that the benefits showed herein are of broad relevance and reinforce the importance of genome assembly projects for other non-model species.
**Corresponding Author:**	Patricio Munoz, Ph.D.<br>University of Florida<br>Gainesville, FLORIDA UNITED STATES
**Corresponding Author Secondary Information:**	
**Corresponding Author's Institution:**	University of Florida
**Corresponding Author's Secondary Institution:**	
**First Author:**	Juliana Benevenuto, Ph.D.
**First Author Secondary Information:**	
**Order of Authors:**	Juliana Benevenuto, Ph.D.
	Luís Felipe Ventorim Ferrão, Ph.D.
	Rodrigo Rampazo Amadeu, Master
	Patricio Munoz, Ph.D.
**Order of Authors Secondary Information:**	
**Response to Reviewers:**	Dear Editor,<br>Thanks for the positive review and comments.<br><br>We agree with the reviewer and with you about adding two more references. Thus, we added text and two references reinforcing that high-quality assemblies also helped in:<br><br>1) Refinement of uniquely mapped reads in resequencing data analysis in Brassica (Lines 74-76)<br>2) resolution of QTL regions underlying relevant traits and detection of targets and design of guide RNAs for genome editing in Wheat (Lines 104-108)<br><br>We limited our addition of references to cases where improvement to breeding-related areas was documented.<br><br>Please accept our revised version.

Powered by Editorial Manager® and ProduXion Manager® from Aries Systems Corporation

Additional Information:	
Question	Response
Are you submitting this manuscript to a special series or article collection?	No
**Experimental design and statistics**<br><br>Full details of the experimental design and statistical methods used should be given in the Methods section, as detailed in our Minimum Standards Reporting Checklist. Information essential to interpreting the data presented should be made available in the figure legends.<br><br>Have you included all the information requested in your manuscript?	Yes
**Resources**<br><br>A description of all resources used, including antibodies, cell lines, animals and software tools, with enough information to allow them to be uniquely identified, should be included in the Methods section. Authors are strongly encouraged to cite Research Resource Identifiers (RRIDs) for antibodies, model organisms and tools, where possible.<br><br>Have you included the information requested as detailed in our Minimum Standards Reporting Checklist?	Yes
**Availability of data and materials**<br><br>All datasets and code on which the conclusions of the paper rely must be either included in your submission or deposited in publicly available repositories (where available and ethically appropriate), referencing such data using a unique identifier in the references and in the "Availability of Data and Materials" section of your manuscript.	Yes

Have you have met the above requirement as detailed in our Minimum Standards Reporting Checklist?	

Powered by Editorial Manager® and ProduXion Manager® from Aries Systems Corporation

Manuscript

Click here to view linked References

Click here to access/download;Manuscript;Benevenuto et al 201x_Commentary_Revised.docx

How can a high-quality genome assembly help plant breeders?

Juliana Benevenuto, Blueberry Breeding and Genomics Laboratory, Horticultural Sciences Department, University of Florida, Gainesville, FL, USA. Email: jbenevenuto@ufl.edu

Luís Felipe V. Ferrão, Blueberry Breeding and Genomics Laboratory, Horticultural Sciences Department, University of Florida, Gainesville, FL, USA. Email: lferrao@ufl.edu

Rodrigo R. Amadeu, Blueberry Breeding and Genomics Laboratory, Horticultural Sciences Department, University of Florida, Gainesville, FL, USA. Email: rramadeu@ufl.edu

Patricio Munoz, Blueberry Breeding and Genomics Laboratory, Horticultural Sciences Department, University of Florida, Gainesville, FL, United States of America. Email: p.munoz@ufl.edu

Corresponding author: Patricio Munoz
Address: 2550 Hull Road, P.O. Box 110690, Gainesville, FL, 32611
Email: p.munoz@ufl.edu

ABSTRACT

The ever-decreasing costs to generate next-generation sequencing data and the significant improvements in *de novo* sequence assemblers have made it possible to obtain reference genomes for most eukaryotes, including relatively minor crops such as blueberry (*Vaccinium corymbosum*). Nevertheless, these genomes are at various levels of completeness and only few have been anchored to chromosome-scale and/or are haplotype-phased. This commentary highlights the impact of a high-quality genome assembly for plant breeding and genetic research by showing how it affects our understanding of the genetic architecture of important traits, aids the marker selection, and the detection of candidate genes. To this end, we compared the results of genome-wide association studies (GWAS) and genomic selection (GS) that were already published using a blueberry draft genome as reference with the results using the recent released chromosome-scale and haplotype-phased blueberry genome. Although this comparison had been motivated by its potential application in blueberry, we believe that the benefits showed herein are of broad relevance and reinforce the importance of genome assembly projects for other non-model species.

Keywords: *Vaccinium*, genome assembly, gene, GWAS, genomic prediction

Background

Assembling plant genomes using short-read-based sequencing is a challenging task, especially because most plant genomes are large, highly repetitive, and have undergone ancient and recent rounds of polyploidization. Thanks to the new sequencing methods, researchers have been able to achieve chromosome-scale haplotype-phased genome assemblies more inexpensively and quickly than previous decades. The cultivated blueberry (*Vaccinium corymbosum*) is an outcrossing tetraploid species (2n=4X=48), and 48 pseudomolecules from the northern highbush cultivar 'Draper' were recently assembled and phased [1]. To accomplish this, the authors used a combination of Illumina paired-end and mate-pair libraries, 10X Genomics Chromium, and Hi-C scaffolding strategies.

One year ago, our group at University of Florida performed GWAS analyses in a southern highbush blueberry (SHB) breeding population in order to detect single nucleotide polymorphisms (SNPs) associated with fruit-related traits [2]. At that time, we used the available draft genome as a reference for SNP calling and gene mining of significant associations. The draft genome assembly was performed for a diploid northern highbush ('W8520') using short-reads from 454 pyrosequencing and Illumina platforms [3,4]. This draft assembly is highly fragmented with 13,757 scaffolds (N50 of 145 kb) and incomplete gene predictions. With the recent release of a new genome assembly by Edger's group at Michigan State University published in *GigaScience* [1], we raised the question: how would a high-quality reference genome affect our previous results and future research? To this end, we re-analyzed our data using nearly the same SHB breeding population, but accommodating changes in probe selection and tetraploid genotype calling that, currently, we believe to be more appropriate (see Figure 1A). The impact of a chromosome-scale and haplotype-phased genome was compared in terms of unique probe alignment, genetic architecture of the traits, candidate gene mining, and genomic prediction.

Selection of probes for targeted SNP calling

A total of 31,063 probes of 120-mer were originally designed based on the 'W8520' genome for targeted capture-seq genotyping by RAPiD Genomics (Gainesville, FL, USA). Probes were designed for enrichment of genic and single mapped genomic regions. The probe

sequences were then aligned against the high-quality ‘Draper’ genome using blastn with e-value threshold of 10^{-10} and identity of 80% [5]. As the genome assembly is haplotype-phased, we were able to distinguish probes that aligned only within homeologous groups, but not among them. Therefore, the new genome allowed us to better filtering uniquely mapped probes, and only half of the original probes (15,663) were further used for targeted SNP calling of the SHB population (Figure 1A). The largest chromosome of each homeologous sets (12 total) from ‘Draper’ genome were used as reference for SNP calling, as described in [2]. Likewise, a high-quality reference genome also refines the proportion of uniquely mapped reads during the alignment of resequencing data as shown in *Brassica* species [6].

Genetic Architecture of the Traits

GWAS can provide the first insights into the genetic architecture of a trait by identifying the number of significant loci, genomic position, mode of gene action, and effect on the phenotypic variation. In this step, the high-resolution positioning of SNPs in the chromosome-scale assembly played an important role in unraveling the genetic architecture of the traits. Using this new pipeline, which also included more accurate genotype calling using *updog* R-package [7], we were able to find significant SNPs with additive gene action mode (e.g., fruit pH), and novel associations (e.g., fruit firmness and scar size) that were not detected in our previous publication [2] (Figure 2B). Moreover, we performed GWAS for a new trait, the volatile geranyl-acetone, extracted and quantified using gas chromatography mass spectrometry, for individuals in the same SHB population. Using the ‘W8520’ genome as reference, the significant SNPs for this volatile were scattered throughout the unplaced scaffolds, leading to a mistaken interpretation that many loci are involved in the trait variation (i.e., polygenic). When using the high-quality ‘Draper’ genome, the significant SNPs converged to a tower like structure in the Manhattan plot (Figure 2C), indicating that, instead of polygenic, there are most likely two genomic regions contributing for the trait variation (i.e., oligogenic).

Candidate gene mining

GWAS also provides candidate genes for subsequent validation. A high-quality genome assembly results in a more complete and accurate prediction of the gene repertoire for candidate gene mining. To exemplify, we looked at the nearest gene of the associated SNPs mentioned in Figure 2A for scar size and pH traits in the 'W8520' genome. Both predicted sequences (*CUFF.54762.1* and *CUFF.14779.1*) were incomplete, and no significant similarity was found in the non-redundant blast protein database. However, for all the nearest genes predicted in 'Draper' genome, we could find orthologs and/or functional annotation (Supplementary Table). Similarly, an improved assembly version of the wheat genome (*Triticum aestivum* L.) allowed the full resolution of a QTL in a region that was disrupted in the previous wheat assemblies due to lack of ordering and incomplete gene predictions [8]. In addition, a more complete annotation of genome features provided a framework for selecting targets and design guide RNAs for editing genes underlying traits relevant for breeding [8].

Genomic Selection

GS has become a new tool in breeding programs, assisting the selection of promising materials and maximizing the genetic gains. For its implementation, a high marker density is required in order to capture most of the linkage information between quantitative trait loci (QTL) and SNPs. However, many studies have been showing that improvements in prediction accuracies reach a plateau afterwards despite the increasing in marker density [9]. Moreover, targeted genotyping costs are driven by the number of probes and the number of flow-cell lanes to sequence the entire assay. Therefore, finding an optimal balance between the number of probes/markers and predictive ability is important for a cost-effective GS implementation. By using the 'Draper' genome, we were able to reduce to half the number of probes, and even though achieve similar predictive abilities for most traits compared with the original number in the 'W8520' genome (Figure 1D). Similar predictive abilities in the same SHB population were also reported by [10] using the 'W8520' genome.

Conclusions

Altogether, we can conclude that investing time and resources to obtain a high-quality reference genome is worthy given the benefits it confers to downstream genetic analyses and in the decision-making process for breeding programs. In the case of blueberry, the benefits were: 1) select a superior set of uniquely mapped probes for GWAS and GS, which will help reduce future targeted genotyping costs since fewer probes are needed; 2) a higher precision about the location, number, and gene action of QTLs, thereby improving our understanding of the genetic architecture of the traits through GWAS analyses; 3) higher chances to find the molecular mechanisms underpinning the trait variation in future studies by being able to explore a more complete gene repertoire; 4) achieve similar genomic predictive ability with fewer genotyping probes. All this will translate in less time and funds for implement marker-assisted and genomic selection in the breeding program, and hopefully achieve higher genetic gains in shorter breeding cycles.

List of abbreviations

GS: genomic selection; GWAS: genome-wide association study; QTL: quantitative trait loci; SNP: single nucleotide polymorphism; SHB: southern highbush blueberry

Availability of data

The data used herein were mostly retrieved from published articles. Specifically, the phenotypic and genotypic data from SHB population can be obtained from [2] at Dyrad Digital Repository (doi accession: 10.5061/dryad.kd4jq6h). The ‘Draper’ genome from [1] can be download at CoGe platform (https://genomevolution.org/coge/GenomeInfo.pl?gid=36464). The ‘W8520’ genome from [3,4] can be download at the QuickLoad site (http://www.igbquickload.org/blueberry/V_corymbosum_scaffold_May_2013/).

Competing interests

The authors declare that they have no competing interests.

Funding

This work was funded by the UF royalty fund generated by the licensing of blueberry cultivars.

Authors’ contributions

JB performed the probe selection, SNP calling, and annotation of the genes. LFVF performed the GWAS analyses. RRA performed the GS analyses. PM supervised and provided overall guidance. JB wrote the manuscript with revision from all authors. All authors read and approved the final manuscript.

Ethics approval and consent to participate

Not applicable

Consent for publication

Not applicable

REFERENCES

1. Colle M, Leisner CP, Wai CM, et al. . Haplotype-phased genome and evolution of phytonutrient pathways of tetraploid blueberry. *Gigascience* . 2019;8:1-15.

2. Ferrão LFV, Benevenuto J, Oliveira I de B, et al. . Insights into the genetic basis of blueberry fruit-related traits using diploid and polyploid models in a GWAS context. *Front Ecol Evol* . 2018;6:1-16.

3. Bian Y, Ballington J, Raja A, et al. . Patterns of simple sequence repeats in cultivated blueberries (Vaccinium section Cyanococcus spp.) and their use in revealing genetic diversity and population structure. *Mol Breed* . 2014;34:675–89.

4. Gupta V, Estrada AD, Blakley I, et al. . RNA-Seq analysis and annotation of a draft blueberry genome assembly identifies candidate genes involved in fruit ripening, biosynthesis of bioactive compounds, and stage-specific alternative splicing. *Gigascience* . 2015;4:1-22.

5. Altschul SF, Gish W, Miller W, Myers EW, Lipman DJ. Basic local alignment search tool. *J Mol Biol* . 1990;215:403-10.

6. Belser C, Istace B, Denis E, et al. . Chromosome-scale assemblies of plant genomes using nanopore long reads and optical maps. *Nat Plants* . 2018;4:879–87.

7. Gerard D, Ferrão LFV, Garcia AAF, Stephens M. Genotyping polyploids from messy sequencing data. *Genetics* . 2018;210:789-807.

8. Appels R, Eversole K, Stein N, et al. . Shifting the limits in wheat research and breeding using a fully annotated reference genome. *Science* . 2018;361:1-13.

9. de Los Campos G, Hickey JM, Pong-Wong R, Daetwyler HD, Calus MPL. Whole-genome regression and prediction methods applied to plant and animal breeding. *Genetics* . 2013;193:327–45.

10. de Bem Oliveira I, Resende MFR, Ferrão LFV, et al. . Genomic prediction of autotetraploids; influence of relationship matrices, allele dosage, and continuous genotyping calls in phenotype prediction. *G3*. 2019;9:1189-1198.

Figure 1. A) Differences in plant material and analytical pipeline using the draft genome ‘W8520’ and the chromosome-scale haplotype-phased genome ‘Draper’. Additional steps not mentioned in this figure were performed according to [2]. **B)** GWAS analyses performed for fruit-related traits (scar size, pH, and firmness) using both genome assemblies and considering Bonferroni threshold of 0.05. **C)** GWAS analyses performed for the volatile geranyl-acetone (CAS 3796-70-1) for individuals from the same SHB population (unpublished data), using both genome assemblies and considering Bonferroni threshold of 0.05. **D)** Predictive abilities for eight blueberry fruit-related traits using 31K probes in the ‘W8520’ draft genome and using 15K selected probes using the ‘Draper’ genome as reference. For genomic prediction, we used GBLUP implemented in the sommer R-package, considering tetraploid inheritance in AGH-matrix R-package, and 30-fold cross-validation by splitting the population in 70% training and 30% testing.

Figure

Click here to access/download;Figure;Figure1_all_oldVSnewgenome.tiff

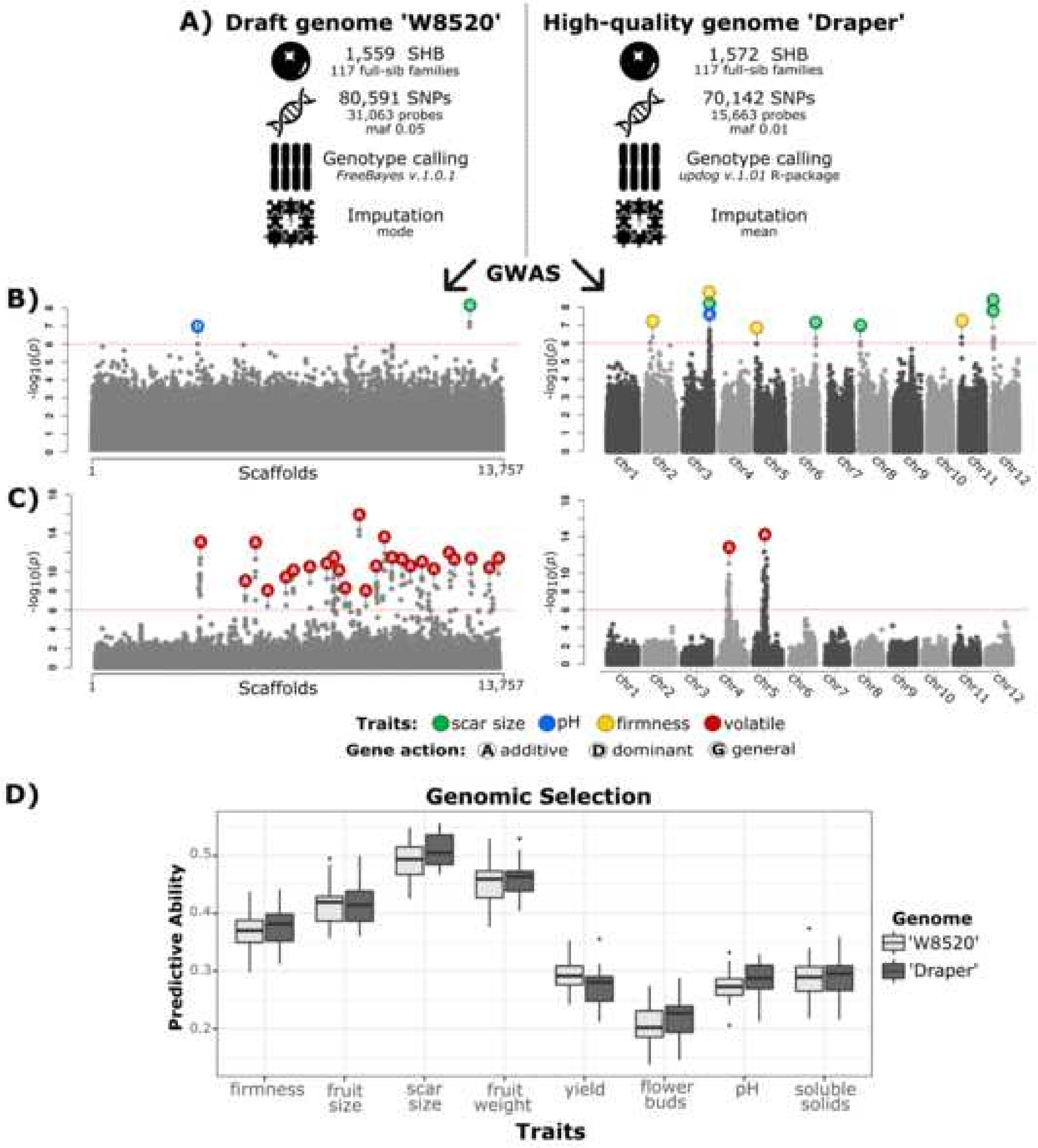

Click here to access/download
Supplementary Material
SupTable_gene_mining_old_newgenome.xlsx
