# Supplementary material for: How can a high-quality genome assembly help plant breeders?
Source: Gigascience. 2019 Jun 10;8(6):giz068. doi: 10.1093/gigascience/giz068 (PMC6558523; doi:10.1093/gigascience/giz068)
Supplement: giz068_Response_to_Reviewer_Comments_Original_Submission [file giz068_response_to_reviewer_comments_original_submission.pdf]

Author's Response To Reviewer Comments

Close

Dear Editor,
Thanks for the positive review and comments.

We agree with the reviewer and with you about adding two more references. Thus, we added text and two references reinforcing that high-quality assemblies also helped in:

1) Refinement of uniquely mapped reads in resequencing data analysis in Brassica (Lines 74-76)
2) resolution of QTL regions underlying relevant traits and detection of targets and design of guide RNAs for genome editing in Wheat (Lines 104-108)

We limited our addition of references to cases where improvement to breeding-related areas was documented.

Please accept our revised version.

Close
